# Investigating the Impacts of Autonomous Vehicles on the Efficiency of Road Network and Traffic Demand: A Case Study of Qingdao, China

**DOI:** 10.3390/s24165110

**Published:** 2024-08-07

**Authors:** Chunguang Liu, Vladimir Zyryanov, Ivan Topilin, Anastasia Feofilova, Mengru Shao

**Affiliations:** 1 Don School, International Education College, Shandong Jiaotong University, Jinan 250357, China; 2 Faculty of Road and Transportation, Don State Technical University, 1 Gagarin sq., Rostov-on-Don 344000, Russia; 3 Urban and Data Science, Graduate School of Advanced Science and Engineering, Hiroshima University, Higashi-Hiroshima 739-8511, Japan

**Keywords:** autonomous vehicles, intelligent transport, transportation, demand, traffic simulation

## Abstract

Rapid urbanization has led to the development of intelligent transport in China. As active safety technology evolves, the integration of autonomous active safety systems is receiving increasing attention to enable the transition from functional to all-weather intelligent driving. In this process of transformation, the goal of automobile development becomes clear: autonomous vehicles. According to the Report on Development Forecast and Strategic Investment Planning Analysis of China’s autonomous vehicle industry, at present, the development scale of China’s intelligent autonomous vehicles has exceeded market expectations. Considering limited research on utilizing autonomous vehicles to meet the needs of urban transportation (transporting passengers), this study investigates how autonomous vehicles affect traffic demand in specific areas, using traffic modeling. It examines how different penetration rates of autonomous vehicles in various scenarios impact the efficiency of road networks with constant traffic demand. In addition, this study also predicts future changes in commuter traffic demand in selected regions using a constructed NL model. The results aim to simulate the delivery of autonomous vehicles to meet the transportation needs of the region.

## 1. Introduction

Due to the continuous rapid development of China’s socioeconomic conditions and the increasing demand for transportation, the number of automobiles has been growing rapidly. This increase in motor vehicle ownership has brought unprecedented pressure to the transportation environment, leading to issues such as traffic congestion and accidents. The development of autonomous vehicles will help alleviate traffic congestion. Human drivers take a long time to react, and communication between human-driven vehicles is also difficult. In traffic flow theory, this is the direct cause of road bottleneck effects. With the increasing density of vehicles in cities, limited environmental awareness and decision-making abilities of drivers will also worsen the levels of traffic congestion. With the application of the autonomous vehicle, with advanced on-board sensors and information and communication equipment, vehicle response time is expected to be improved, the distance between vehicles will be shortened, and braking time will also be reduced. These changes will bring a smoother ride experience and less congestion. The development of the autonomous vehicle will overturn the existing vehicle ownership mode and reduce the waste of Earth’s resources [1,2]. The global theoretical evaluation indicates that, even during the busiest times on urban roads, only 12% of vehicles are in motion at any given time, which means that at least 88% of vehicles are idle during everyday life [3,4]. Furthermore, it is estimated that 95% of the average lifespan of a vehicle is spent idle, with only 5% of driving time still having a significant proportion of idle time [5]. Over the years, a considerable amount of Earth’s resources have been wasted on storing and maintaining these idle vehicles. If vehicles achieve autonomous driving, passengers would not need to own their own vehicles. This means they would only have to pay for mileage services, rather than the cost of the vehicle itself. Establishing a car-sharing model would roughly equalize the demand for vehicles with the number of vehicles set aside, thus saving significant natural and social resources [6,7,8].

The integration of self-driving vehicles (AVs) and connected autonomous vehicles (CAVs) is poised to revolutionize how we use our roads, greatly enhancing both capacity and efficiency of traffic. Various studies, such as those by Lu et al. [9], Guériau and Dusparic [10], and Mavromatis et al. [11], have shown that, as the number of AVs increases, urban traffic flows more smoothly, with less congestion and higher speeds.

Moreover, transitioning to AVs and CAVs offers substantial environmental perks. Research by Kopelias et al. [12] indicates that CAVs can reduce CO_2_ emissions by up to 94% and cut fuel consumption by as much as 90%. Stogios et al. [13] also found that AVs improve emissions and traffic flow, even under aggressive driving conditions.

Safety is another key benefit. Higher rates of CAVs on the road can enhance safety, as shown by Ye and Yamamoto [14], who found that cautious driving strategies of CAVs lower the risk of accidents. Yu et al. [15] suggested that creating AV-only lanes can further boost efficiency and safety, particularly when AV penetration is still growing.

The spread of AVs and CAVs will also profoundly impact urban planning and land use. Gavanas [16] highlighted that AVs demand a rethink of land use, infrastructure, and urban development. Soteropoulos et al. [17] noted that, while private AVs could lead to more miles traveled and less public transport use, shared AV fleets might reduce the need for parking spaces.

However, as AV and CAV technologies rapidly advance, robust policies and regulations are essential. Ahmed et al. [18] pointed out the need for updated laws to keep pace with technological developments, including advanced driving assistance systems (ADAS). Faisal et al. [19] emphasized that policymakers must prepare for the disruptive potential of AVs with comprehensive frameworks and interventions.

In terms of social equity, AVs also hold promise. Cohn et al. [20] found that scenarios involving high-occupancy AVs and improved transit can offer significant benefits, such as closing gaps in job accessibility and travel efficiency for disadvantaged populations.

Continuous research and simulation studies are crucial to understand and navigate the broader impacts of AVs and CAVs. Guériau and Dusparic [10], Fakhrmoosavi et al. [21], Chen et al. [22], and Gurumurthy et al. [23] underscored the importance of using simulation tools to model these impacts on traffic flow, safety, and efficiency, and to examine how different types of vehicles and driver behaviors interact.

In the field of contemporary research on travel behavior and demand forecasting, there is a strong emphasis on the utilization of advanced modeling techniques, the incorporation of comprehensive socioeconomic and trip-related data, the practical application of policy, and the continuous development of innovative methodological approaches [11,14,24].

Nested logit (NL) models are frequently employed in the analysis of travel behavior and the forecasting of demand [25,26,27] because they are effective at capturing complex correlation patterns and hierarchical decision-making processes that are challenging for standard multinomial logit (MNL) models to accommodate. For example, Hess et al. [28] employed a cross-nested logit model to investigate the correlations between vehicle and fuel type choices, while Dissanayake and Morikawa [29] used an NL model to examine the relationship between household vehicle ownership and mode choice in Bangkok.

The objective of researchers in this field is to develop and calibrate behavioral models that can be used to predict travel demand and to evaluate the impacts of transportation policies. These models frequently integrate revealed preferences (RP) and stated preferences (SP) data in order to enhance the accuracy of the results. For instance, Bierlaire and Thémans [30] constructed models to predict drivers’ responses to real-time traffic information in Switzerland, while Ghader et al. [31] proposed a copula-based continuous cross-nested logit model for scheduling tours in activity-based travel models.

It is of great importance to incorporate socioeconomic factors, household characteristics, and trip specifics into the analysis, as these elements have a significant influence on mode choice and travel behavior. In their study of commuting trips in Xi’an, China, Ma et al. [32] incorporated factors such as household income, commuting distance, and occupation. Similarly, Shang and Zhang [33] employed a nested logit model to examine the travel mode choices of residents, taking into account a comprehensive range of influencing factors. These studies frequently extend to practical applications, including policy analysis and strategic planning for congestion reduction, environmental impact, and infrastructure development. For example, Dissanayake and Morikawa [29] employed their model to analyze congestion-reduction policies in Bangkok, while Elmorssy and Tezcan [34] introduced a novel modeling approach to enhance travel demand forecasting and its policy implications.

Notable advancements have been made in the field of traffic simulation platforms, particularly the Simulation of Urban Mobility (SUMO), which now offer robust support for autonomous vehicle (AV) testing. Kusari et al. [35] enhanced SUMO’s user experience and traffic variability by calibrating the intelligent driver model (IDM) and integrating SUMO with OpenAI gym to create a Python 3.10 package for real-world simulations. In a further development, Li et al. [36] introduced a novel framework combining SUMO with CARLA to simulate complex environments, thereby enhancing the perception capabilities of AVs through realistic sensor outputs. In a related study, Shi et al. [37] investigated the integration of intelligent transportation systems (ITS) with AVs, demonstrating that increased AV penetration can significantly reduce average trip durations and delays.

A number of studies have concentrated on the effect of AVs and connected autonomous vehicles (CAVs) on traffic flow, safety, and efficiency in mixed traffic scenarios. In their review of traffic simulators, Vrbanić et al. [38] evaluated the suitability of various platforms, including VISSIM, AIMSUN, and SUMO, for use with network simulators. They found that AIMSUN was better suited to less complex models, while VISSIM demonstrated greater capabilities for more complex scenarios. Kavas-Torris et al. [39] employed SUMO to examine the impact of AVs on roadway mobility, demonstrating that augmented levels of autonomy enhance mobility for both AVs and non-AVs, albeit at lower speeds for the latter. In a recent study, Andreotti and Pinar [3] examined the transition from fully autonomous to partially autonomous traffic, highlighting improvements in safety and efficiency due to the introduction of AV features.

Furthermore, research has been conducted with the objective of optimizing traffic flow and energy efficiency through the implementation of various strategies. Wen et al. [40] developed a real-time control model for CAVs at signal-controlled intersections, which resulted in a notable reduction in energy consumption without any adverse effects on traffic efficiency. Mushtaq et al. [41,42] proposed a two-level approach combining platooning and collision avoidance strategies with the aim of managing AV traffic flow, and their findings demonstrated a considerable improvement in performance when this approach was simulated.

Therefore, it is found that a large amount of research is focused on the safety, cost, efficiency, and infrastructure aspects of autonomous driving vehicles. A few studies are based on the potential impact and outcomes of autonomous driving vehicles on urban land use and urban form. There is little research on whether autonomous driving vehicles can meet the transportation needs of different cities.

## 2. Materials and Methods

In this paper, a nested logit (NL) model was used to predict demand for unmanned transport. The nested logit model is a popular alternative to the traditional multinomial logit (MNL) model for modeling consumer preferences and choice behavior because the traditional MNL model falls under the umbrella of probabilistic choice models and offers an advantage by accounting for the correlation in unobserved factors that affect the utility of different alternatives. In addition, the traditional MNL model is based on the key assumption of ‘independence from irrelevant alternatives’ (IIA). This means that the odds of choosing one option over another are not affected by the introduction or removal of a third option [43]. However, this assumption often fails in typical scenarios. The NL model addresses this issue by grouping similar alternatives. Therefore, the nested logit model is a valuable tool in transportation planning, market research, healthcare, and public policy due to its flexible structure, which provides greater flexibility than MNL models. Its ability to predict consumer behavior based on factors such as travel time, cost, comfort, and environmental impact, grouped under different ‘nests’, is essential in these areas. It is common for alternative modes of transport to have similarities, which can lead to correlation of the error terms of the utility functions in the choice set. For instance, the level of comfort when traveling by private car may be more similar to that of traveling by taxi than to that of traveling by bus. The NL model groups similar alternatives into ‘nests’ to account for correlations and produce more accurate estimates of travelers’ choices than the traditional multinomial logit (MNL) model, which assumes that alternatives are independent.

Based on the utility maximization theory, this study assumes that the consumers in the NL model aim to achieve the highest level of satisfaction or benefit possible from the products or services they consume, given their limited resources. Consumer behavior is mathematically represented through utility functions that account for their preferences and trade-offs, even when there are resource constraints. In the literature, a standard NL model operates in two stages. During the initial stage, a consumer narrows down their options to a ‘nest’ which is a subset of alternatives. In the subsequent stage, the consumer makes a decision within this nested set of alternatives. Each stage reflects a set of conditions that influence the decision-making process.

This study focuses on the planned development of Qingdao City in 2030. Qingdao is situated in the eastern part of China, specifically the southeastern region of the Shandong Peninsula and the northern part of the Yellow Sea. The study area encompasses the Old Town District (the central part of Qingdao), the South District, the Central District, and the North District.

**Figure 1:**
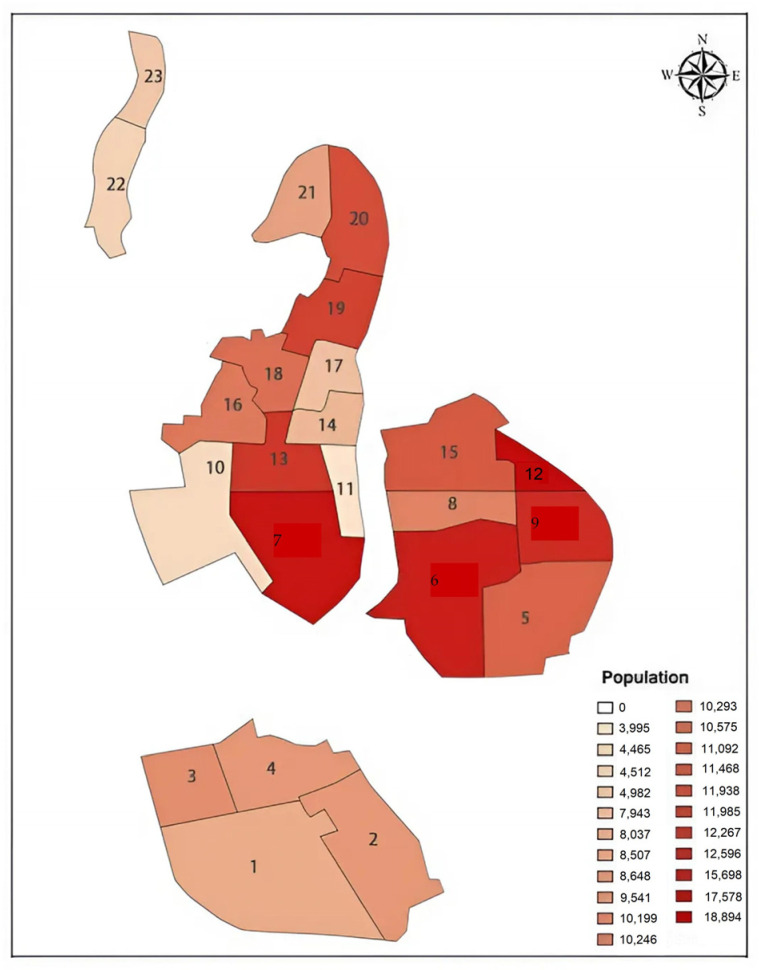
Population distribution of Qingdao.

First, the population distribution in the Old Town area was projected. The Old Town area has a population of about 107,800 and is divided into 11 transportation districts, and the distribution of residential population in each transportation district is shown in Figure 1.

Although utility maximization is the foundation of the theory, the model’s accuracy and outcome are significantly influenced by the factors used to construct it, such as costs, preferences, and constraints. Therefore, it is crucial to carefully select these factors to accurately represent the set of alternatives and consumer behavior. Taking the 16th region on the map as an example, this region has a population of 10,293 residents. The residents in this region have 22 working area choices and 6 commuting mode choices, resulting in 132 commuting choices for the residents in this region. For the rest of the territory, the distribution of 127,200 residents can be inferred from the corresponding probabilities.

Using an NL model, we can predict and compare driving patterns in two scenarios: (1) without autonomous vehicles and (2) with autonomous vehicles. In the scenario without autonomous vehicles, residents primarily use non-motorized vehicles, buses, and cars. In the scenario with autonomous vehicles, we examine residents’ commuting patterns. Travel modes mainly include non-motor vehicles, public transport, shared autonomous vehicle, private autonomous vehicle, and private cars. Private cars require parking fees.

(1) Scenario 1: without autonomous vehicles

In this scenario, residents primarily use non-motorized vehicles, buses, and cars. The nested logit model structure for travel mode choice can be represented as follows:

**Top Level**: Travel Mode Choice

➢ **Sub-level 1**: Non-Motorized Vehicles: Cycling
➢ **Sub-level 2**: Motorized Vehicles
  **Sub-level 2.1**: Public Transport: Bus
  **Sub-level 2.2**: Private Transport: Car

Travel mode choice structure captures the hierarchical decision-making process where residents first decide whether to use motorized or non-motorized transport. Within each category, they further choose specific modes.

Below are the nested logit (NL) utility functions, considering that sublevels 1 and 2 are nests. The NL model groups similar alternatives into nests as follows:

**Top Level**: Travel Mode Choice

➢ **Nest 1**: Non-Motorized Vehicles (M1): Cycling (M11)
➢ **Nest 2**: Motorized Vehicles (M2)
  **Sub-level 2.1**: Public Transport (M21): Bus (M211)
  **Sub-level 2.2**: Private Transport (M22): Car (M221)

The utility of choosing mode *i* within nest j can be formulated as follows:

*U_ij_* = *V_ij_* + *ε_ij_* (1)

where *V_ij_* is the deterministic component of utility for mode *i* within nest *j*, and *ε_ij_* is the random error term. In addition, the deterministic component includes various factors that influence the traveler’s choice, typically modeled as:

*V_ij_* = *β*_0_ + *β*_1_*C_ij_* + *β*_2_*T_ij_* + *β*_3_*S_ij_* (2)

where *C_ij_* is the travel cost for mode *i* within nest *j*, *T_ij_* is the travel time for mode *i* within nest *j*, *S_ij_* represents socioeconomic characteristics influencing the utility (cost of housing, distance to public services, and other conditions).

Inclusive value (IV) for each nest *j* can be modeled as:

*IV_j_* = ln(∑_*i*∈*j*_ e^*μVij*^) (3)

where *μ* is the nesting parameter (0 < *μ* ≤ 1). Then, the probability of choosing mode *i* within nest *j* is calculated as:

*P_ij_* = *P* (*i*∣*j*)⋅*P* (*j*) (4)

where the conditional probability *P* (*i*∣*j*) is

*P* (*i*∣*j*)= e^*μVij*^/∑_*k*∈*j*_ e^*μVkj*^ (5)

In addition, the marginal probability *P* (*j*) is

*P* (*j*) = e^*λIVj*^/∑_*m*_ e^*λIVm*^ (6)

where *λ* is the scale parameter (usually set to 1). Nesting parameter (*μ*) accounts for the degree of similarity among alternatives within the same nest. It ranges between 0 and 1, where values closer to 1 indicate higher similarity (more correlation) among the nested alternatives. Scale parameter (λ) is typically set to 1; this parameter ensures that the model is scale-consistent across different levels of the hierarchy [44].

(2) Scenario 2: with autonomous vehicles

In this scenario, travel modes include non-motorized vehicles, public transport, shared autonomous vehicles, private autonomous vehicles, and private cars. The nested logit model structure can be represented as follows:

**Top Level**: Travel Mode Choice

➢ **Sub-level 1**: Non-Motorized Vehicles: Cycling
➢ **Sub-level 2**: Motorized Vehicles
  **Sub-level 2.1**: Public Transport: Bus
  **Sub-level 2.2**: Autonomous Vehicles: Shared AV (SAV) and Private AV (PAV)
  **Sub-level 2.3**: Private Transport: Traditional Car

The model distinguishes between shared and private autonomous vehicles and captures the decision-making process within each category. Residents first decide whether to use motorized or non-motorized transport. Within motorized transport, they further decide between public transport, autonomous vehicles, and traditional cars.

Below are the nested logit (NL) utility functions, considering that sublevels 1 and 2 are nests. The NL model groups similar alternatives into nests as follows:

**Top Level**: Travel Mode Choice

➢ **Nest 1**: Non-Motorized Vehicles (M1): Cycling (M11)
➢ **Nest 2**: Motorized Vehicles (M2)
  **Sub-level 2.1**: Public Transport (M21): Bus (M211)
  **Sub-level 2.2**: Autonomous Vehicles (M22): SAV (M221) and PAV (M222)
  **Sub-level 2.3**: Private Transport (M22): Traditional Car (M231)

The utility of choosing mode *i* within nest *j* is determined in Equation (1) and the deterministic component includes various factors that influence the traveler’s choice, typically modeled as:

*V_ij_* = *β*_0_ + *β*_1_*C_ij_* + *β*_2_*T_ij_* + *β*_3_*S_ij_* + *β*_4_*AV_ij_* (7)

where *C_ij_* is the travel cost for mode *i* within nest *j*, *T_ij_* is the travel time for mode *i* within nest *j*, *S_ij_* represents socioeconomic characteristics influencing the utility (cost of housing, distance to public services, and other conditions), *AV_ij_* is a dummy variable indicating whether the mode is an autonomous vehicle. Other key components of the nested logit model are determined according to (2)–(6).

**Figure 2:**
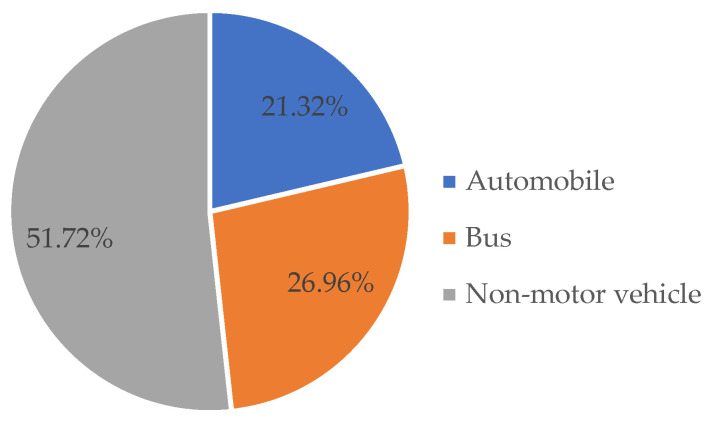
Driving scenario without autonomous vehicle.

Based on the population preferences and pattern selection, the NL model predicts the distribution of traffic and mode proportions in the given area under autonomous driving. The mode proportion or mode share refers to the percentage of travelers who choose a particular mode of transportation during their trips. By multiplying the vehicle occupancy rate of autonomous vehicles with the generated and attracted traffic flows in unit traffic volume, we can determine the share of vehicles on the road for each trip. In the absence of autonomous vehicles, non-motorized vehicles account for the largest proportion of commuting, followed by buses, with private cars accounting for 21.31% (Figure 2).

**Figure 3:**
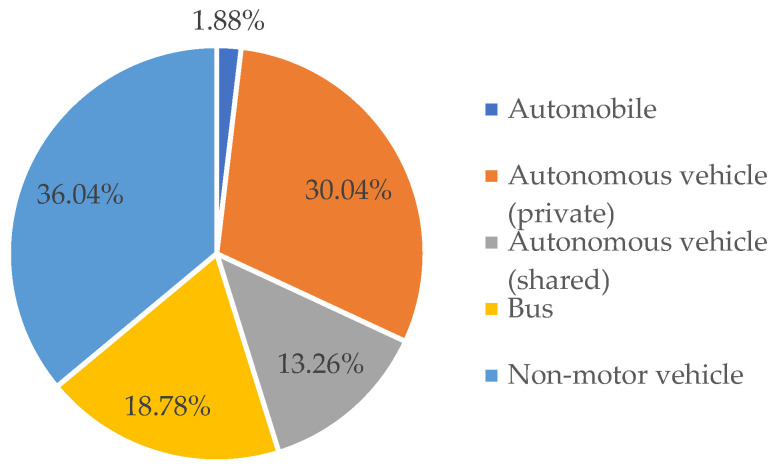
Driving modes in autonomous driving scenarios.

In the context of autonomous driving, the percentage of people who use cars as their commuting mode has increased to 45.18% (Figure 3). Among the group of people who choose autonomous driving vehicles as their commuting mode, private autonomous driving vehicles have become the preferred option, accounting for 30.04% of the vehicle share, while shared autonomous driving vehicles account for 13.26%.

**Figure 4:**
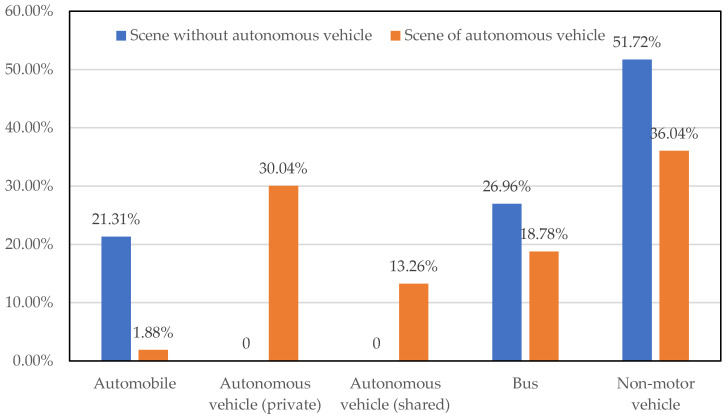
Changes in commuting patterns.

The changes in each commuting mode are illustrated in Figure 4. Due to the significant impact of parking fees on residents’ choice of transportation, the increase in parking fees for private cars in the autonomous driving scenario has caused the percentage of private cars to decrease from 21.31% to 1.88%. In addition to the initial group of car commuters, autonomous driving vehicles will also attract a portion of the public transportation commuters, especially those who do not normally use cars for commuting. Approximately 15.69% of non-car commuters and 8.18% of public transportation commuters choose to use autonomous driving vehicles for their daily commute.

Road network and predicted vehicle ratios are imported into the traffic modeling software SUMO 1.19.0 for traffic assignment, completing the traffic demand prediction. Simulation of Urban Mobility (SUMO) is an open-source, highly portable, microscopic, and continuous road traffic simulation package designed to handle large road networks. It allows for the implementation of complex traffic management schemes, enabling the creation of a rich set of intermodal traffic management solutions. In this study, the traffic assignment in SUMO could be divided into four essential steps as follows [45,46]:

1. Network import: Loading the geographic data of the area to be simulated involves using SUMO, which supports a variety of input formats such as XML and OpenStreetMap OSM files. In addition, it allows for defining roads, intersections, lanes, and traffic lights in the network.
2. Demand modeling, which involves representing user behavior in the model. This could include different modes of transportation and routes. SUMO provides a range of tools for creating and managing various demand models.
3. The simulation is then executed, enabling the user to observe the traffic as it develops over time. Following the simulation, a variety of metrics can be gathered for analysis, such as travel times, route choices, and emissions. SUMO offers various tools to aid analysis, such as plotting tools and export functions.
4. Traffic assignment in SUMO is managed through its ‘route choice models’, which can be explained using the widely used nested logit model for network assignment. SUMO is a valuable tool for transport planning. Traffic assignment can provide planners with an understanding of the anticipated road usage under various conditions or transport policies.

The standard SUMO parameters have been adjusted to simulate a possible future scenario for autonomous vehicles. This study utilized the default car-following model known as the Krauss model. This parameter tuning specifically focused on the longitudinal dynamics involving acceleration, deceleration, and gap acceptance. These driving behaviors were defined and fine-tuned as key parameters within the car-following framework of SUMO (refer to Table 1). The modified model aimed to enable vehicles to travel at maximum safe speeds while ensuring constant safety measures are in place, ensuring avoidance of collisions by allowing for braking within predefined acceleration limits between the leading and following vehicles. Here is a breakdown highlighting the customizable parameters within the Krauss car-following model:

- Mingap: the offset to the leading vehicle when standing in a jam (in m).
- Accel: the acceleration ability of vehicles of this type (in m/s^2^).
- Decel: the deceleration ability of vehicles of this type (in m/s^2^).
- Emergency decel: the maximum deceleration ability of vehicles of this type in case of emergency (in m/s^2^).
- Sigma: the driver imperfection (between 0 and 1).
- Tau: the driver’s desired (minimum) time headway (reaction time) (in s) [9].

## 3. Road Network Modeling Results

To investigate the deployment of an appropriate number of unmanned vehicles to meet the traffic demand of selected regions, this study modeled the scenario of the penetration rate of unmanned vehicles and assumed that the morning peak traffic demand would remain unchanged. The construction of the modeling scenario was the first step. The road network used in this experiment is a component of the planned road network for the central city of Qingdao in 2030. The traffic flow represents the projected traffic demand for this planning area during the morning rush hour, from 8:00 to 9:00. While unmanned vehicles can transmit information wirelessly, traditional traffic signs and signal lights may still be necessary to consider for long-term coexistence with human-driven vehicles. The micro-simulation experiment primarily investigates the coexistence of unmanned and man-made vehicles on an urban scale. The experiment excludes irrelevant factors, such as the geometric design of the road in the unmanned vehicle scenario and the location of charging equipment. It only considers the impact of unmanned vehicles with varying permeability on traffic efficiency and their ability to meet traffic demand when controlling signal lights. The experiment’s independent variable is the proportion of unmanned vehicles in traffic flow. The following values are used: 5%, 10%, 20%, 20%, 30%, 40%, 50%, 60%, 70%, 70%, 80%, 90%, and 100%. The dependent variable is urban transport efficiency, and the evaluation indicators include average kilometers traveled, average delay time, average waiting time, average travel time, and average speed (refer to Table 2). To increase the accuracy of the modeling results, this study conducted five simulations for different permeability scenarios, replacing the random speed each time. It should also be noted that data with large errors were removed and the average was calculated for better analyses.

**Figure 5:**
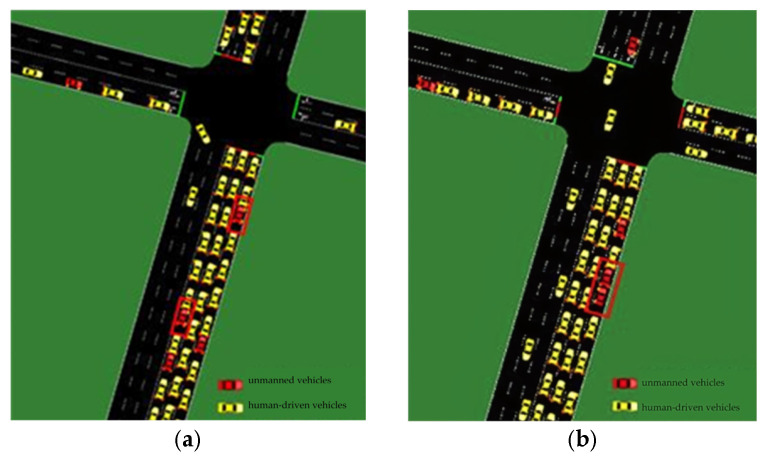
Visualizations of the simulation scenario for the rate of unmanned vehicles in the traffic flow of 10% (**a**) and 20% (**b**).

The road network and peak hour OD data for the selected areas were obtained from the ‘Qingdao Urban Traffic Management Planning Project’. The peak hour OD data were obtained from the 2030 land use plan and local population projections for the selected areas. Analysis of the full simulation model results indicates that unmanned vehicles can effectively improve traffic efficiency and better meet local transport needs when operating in groups. As the penetration rate increased from 0% to 10%, the average delay, average waiting time, and average traveling time increased, while the average travel speed decreased. Observing the modeling process revealed that, as the proportion of unmanned vehicles in traffic flow increased from 0% to 10%, the number of human-driven vehicles remained high. Additionally, most unmanned vehicles appeared singly on the road network (see Figure 5a). This resulted in reduced traffic efficiency, as the unmanned vehicles cannot be utilized to improve the overall traffic flow and may even interfere with human-driven vehicles.

As the penetration rate increased from 10% to 20%, the number of unmanned vehicles operating in the same group on the road network also increased, as shown in Figure 5b. Currently, unmanned vehicles can communicate with each other using vehicle communication. This allows them to accurately adjust their speed based on the preceding vehicle, reducing the following distance and improving traffic efficiency. As a result, the delay time, waiting time, and travel time of unmanned vehicles tended to decrease, while their operating speed tended to increase; however, as human-driven vehicles still made up most vehicles on the road network, their performance has remained largely unchanged.

**Figure 6:**
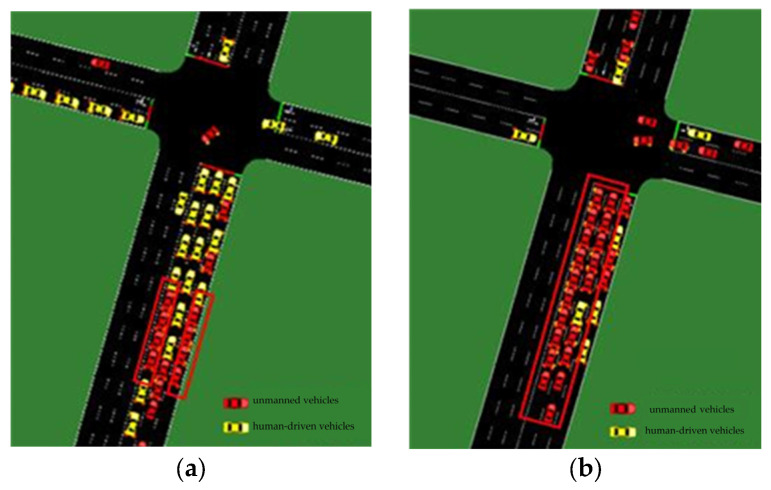
Visualizations of the simulation scenario for the rate of unmanned vehicles in the traffic flow ((**a**) 50% and (**b**) 80%).

When the proportion of unmanned vehicles in the traffic flow increased from 20% to 50%, the likelihood of multiple unmanned vehicles forming a group also increased (see Figure 6a). This resulted in longer average delay times, waiting times, and travel times for unmanned vehicles. The delay, waiting, and travel times of human-driven vehicles decreased as unmanned vehicles improved the traffic efficiency of the road network to a certain extent.

As the proportion of unmanned vehicles in the traffic flow increased from 50% to 100%, the number of unmanned vehicles in the scene exceeded that of human-driven vehicles (refer to Figure 6b). This resulted in a decrease in average delay time, average waiting time, and average traveling time, and a significant increase in traffic speed. Please note that the yellow vehicles in the picture represent human-driven vehicles, while the red ones represent unmanned vehicles.

However, unmanned vehicles had higher traffic efficiency than human-driven vehicles. When the proportion of unmanned vehicles in the traffic flow was below 20%, overall traffic efficiency did not significantly improve. When the proportion of unmanned vehicles in traffic flow exceeded 20%, overall traffic efficiency began to improve. When more than 50% of the vehicles in traffic were unmanned, the probability of group operation of these vehicles increased, leading to improved urban traffic efficiency. This study’s results indicate that, if the number of unmanned vehicles launched in the early stages is small, the efficiency improvement of the entire system will be limited. Only when the proportion of unmanned vehicles launched exceeds 20% can overall traffic efficiency be greatly improved, and people’s transport needs can be satisfied.

**Figure 7:**
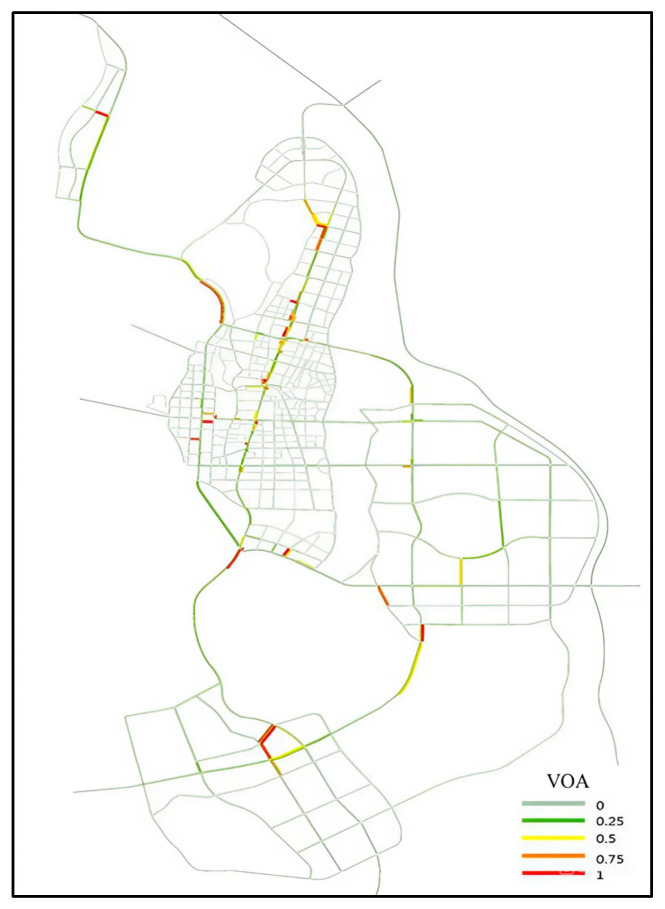
Lane occupancy during simulation.

The Report on Development Forecast and Strategic Investment Planning Analysis of China’s autonomous vehicle industry states that the current development scale of China’s intelligent autonomous vehicles has exceeded market expectations [47]. Therefore, in this scenario for Qingdao, the penetration rate of autonomous vehicles is 90%, and the lane occupancy rate of the road network during the simulation process is shown in Figure 7. The simulation results are shown in Table 3.

Simulation results indicate that, although the market share of autonomous vehicles in the traffic flow is 90% in the predicted scenario, the average speed of vehicles is lower. There are two reasons for this result: First, the increase in demand for vehicles. Autonomous vehicles not only replace human-driven vehicles but also attract some passengers who commute using other modes of transportation, increasing the traffic flow during commuting. The second reason is the increase in average commuting distance, which can be seen from the more congested intersections between regions during the simulation process. In the autonomous driving scenario, due to the higher efficiency of autonomous vehicles compared with human-driven vehicles, the accessibility of surrounding areas increases, and residents tend to choose residential buildings with excellent public services, resulting in increased travel distance. This leads to an increase in traffic flow and severe congestion on connecting roads between regions.

**Figure 8:**
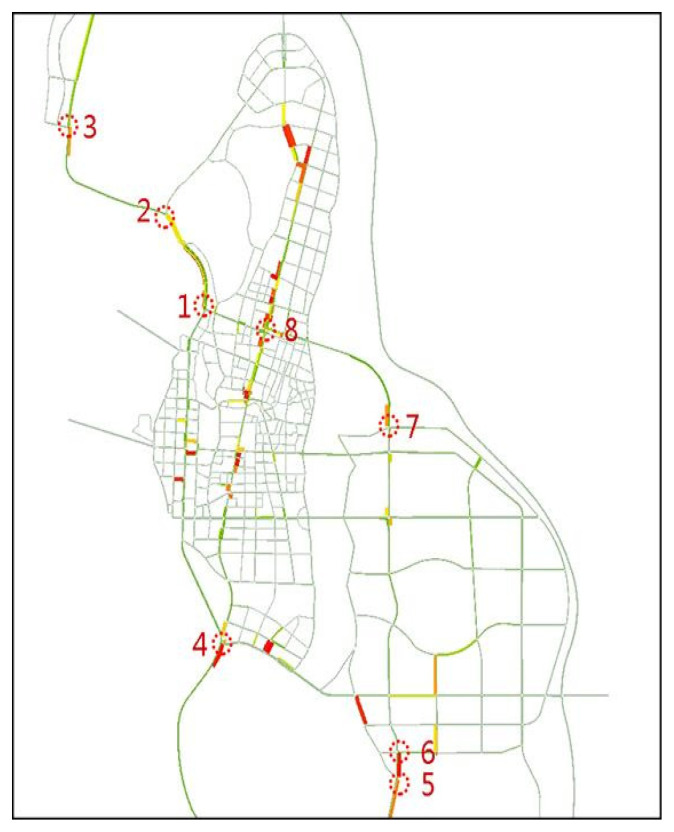
Location of apparently congested nodes.

In the road network, there are eight distinct congested nodes, as shown in Figure 8. Nodes 1–7 are nodes connecting the area to the urban corridor, while Node 8 is an important node for north-south and eastward traffic.

In our paper, Qingdao Districts 16 and 22 (see Figure 1) and the road connecting them were selected for detailed consideration. Therefore, the simulation results’ data were compiled for intersections 1, 2, 3, and 8. In the simulation model, the average queue length for each entrance of several intersections was calculated. Additionally, under the same traffic demand, a scenario was simulated where the share of autonomous vehicles in the traffic flow was 0%, and a comparative analysis was conducted.

Intersection 1: Intersection 1 is located in the northern part of the designated area and is one of the important external exits in the area. The average queue length at the intersection is shown in Table 4. The names in the headings correspond to the direction of traffic on the lanes. In the scenario where autonomous vehicles account for 0% of the traffic flow, the queue length at each entrance exceeds the 90% scenario. In the scenario where autonomous vehicles account for 90% of the traffic flow, the queue length for left turns at the northbound entrance is 304.07 m, and the queue length for straight through traffic is 75.32 m. In the 0% scenario, the queue length for left turns at the northbound entrance is 791.40 m, and the queue length for straight through traffic is 218.69 m. The intersection is congested but still able to meet the traffic demand.

**Figure 9:**
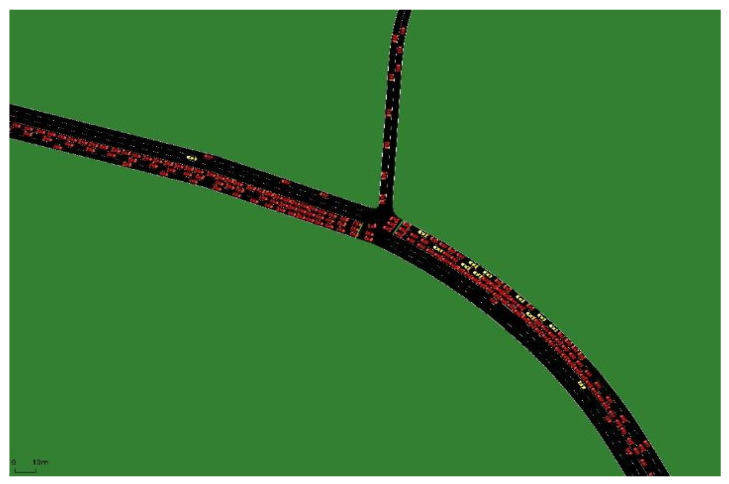
Intersection 2 (scenario with 90% autonomous vehicle share of traffic flow).

Intersection 2 (Figure 9): The queue lengths at Intersection 2 are shown in Table 5. The names in the headings correspond to the direction of traffic on the lanes. In scenarios where autonomous vehicles account for 0% of the traffic flow, the queue lengths are longer compared with scenarios where autonomous vehicles account for 90% of the traffic flow. In the scenario where autonomous vehicles account for 90% of the traffic flow, the queue length for northbound left turns is the longest, approximately 105.86 m; the queue length for southbound through traffic is the longest, approximately 49.64 m. In the scenario where autonomous vehicles account for 0% of the traffic flow, the queue lengths for northbound and eastbound are the longest, as well as the southern direction.

Intersection queue lengths are shown in the table below.

**Figure 10:**
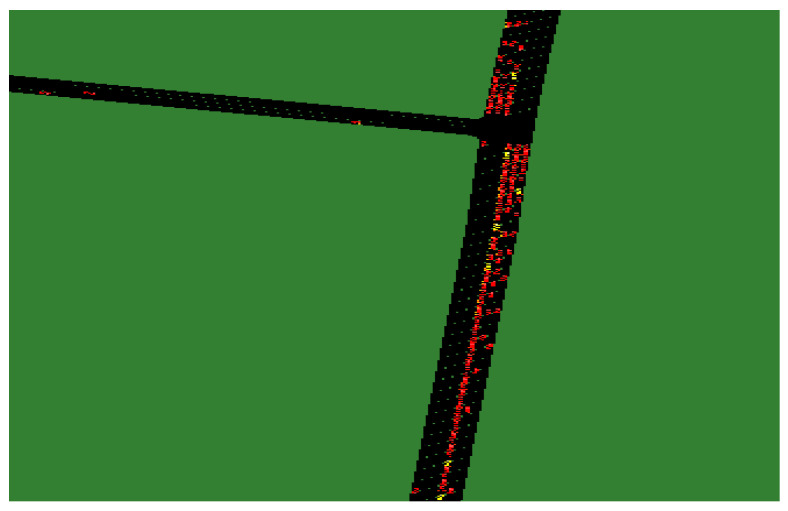
Intersection 3 (90% of autonomous vehicle in the traffic flow).

Intersection 3: Intersection 3 is the outbound exit for the southbound area (Figure 10). The queue lengths at Intersection 3 are shown in Table 6. In the scenario where autonomous vehicles account for 90% of the traffic flow, the average queue length for the southbound entrance is 342.87 m, with a direct queue length of 42.02 m. The average queue length for the northbound entrance is 57.6 m. In the scenario where autonomous vehicles account for 0% of the traffic flow, the queue length for the left lane of the southbound direction is 280.1 m, the queue length for the straight lane is 36.07 m, and the queue length for the southbound straight lane is 43.92 m. The average queue lengths for Intersection 3 are summarized in Table 6. The names in the headings correspond to the direction of traffic on the lanes.

The three lanes of traffic at the south entrance to Intersection 3 are one straight left lane and two straight lanes, and, with the increase in left-turning traffic at the south entrance, these lanes are not set up properly, resulting in longer queues for left turns, with long queues of turning vehicles and left-turning vehicles waiting to enter the left-turn lane in the straight lane adjacent to the straight left lane.

**Figure 11:**
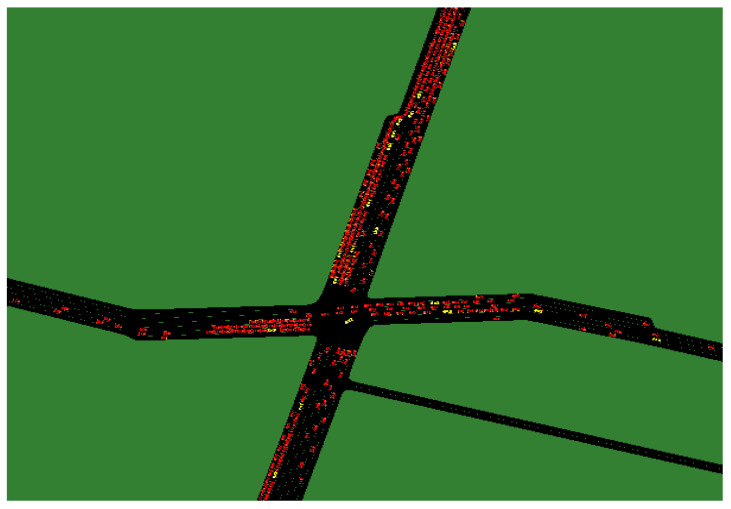
Intersection 8 (90% of autonomous vehicle in the traffic flow).

Intersection 8: Intersection 8 (Figure 11) is the traffic hub for the entire area and has the highest traffic load.

At the intersection of the eighth street, there are five north-south entrance lanes and four east-west entrance lanes. The average queue lengths for the north-south and east-west entrances are shown in Table 7 and Table 8, respectively. In a scenario where self-driving vehicles account for 90% of the traffic flow, the straight queue lengths for northbound and westbound directions will be longer. In a scenario where self-driving vehicles account for 0% of the traffic flow, the queue lengths for northbound straight, westbound straight, and eastbound left turns will be longer.

The main conclusions drawn from this study are as follows:

(1) The observed improvement in traffic efficiency resulting from the introduction of autonomous vehicles (AVs) in a range of contexts can be attributed to a number of key factors. The engineering of AVs is designed to optimize driving patterns, maintain consistent speeds and minimize abrupt stops and starts, which collectively contribute to a more fluid traffic flow. This optimization results in a reduction in overall congestion and an enhancement in average traffic speeds. Furthermore, AVs are equipped with the ability to communicate with one another and with traffic infrastructure, thereby facilitating more efficient coordination at intersections and during lane changes. The inter-vehicle communication system reduces the necessity for unnecessary braking and acceleration, which are typical of human drivers due to their delayed reactions and inconsistent driving behaviors. As a result, the introduction of AVs has resulted in a reduction in average waiting times and travel times. Nevertheless, the relatively modest reduction in average delay times is predominantly attributable to the limited number of traffic lights at urban intersections, which continue to impose constraints on AVs. This highlights the necessity for further integration of AV technology with advanced traffic management systems.
(2) It has been demonstrated that, when the proportion of AVs within the traffic flow is below 20%, there is no significant improvement in overall vehicle efficiency. This is due to the fact that a limited number of AVs are unable to significantly impact the prevailing traffic dynamics, which continue to be largely shaped by human-driven vehicles. Nevertheless, even at lower penetration rates, AVs demonstrate superior efficiency compared with human-driven vehicles, due to the optimized driving algorithms and enhanced adherence to traffic regulations inherent to their design. As the proportion of AVs in the traffic flow exceeds 20%, their impact on traffic flow becomes more pronounced. The inherent communication and coordination capabilities of AVs begin to generate notable improvements in traffic efficiency, leading to a reduction in congestion and an enhancement in average travel speeds. As the proportion of AVs exceeds 50%, the benefits become even more significant. The increased presence of AVs leads to more consistent and predictable traffic patterns, which further improve traffic flow and better meet transportation needs. This threshold represents a critical tipping point, where the collective behavior of AVs can markedly alter traffic conditions, emphasizing the importance of reaching and surpassing this critical mass for optimal efficiency gains.
(3) In scenarios where the proportion of autonomous vehicles (AVs) is significant, an increase in the population and commuting distances will consequently lead to an increase in traffic volume. The potential of autonomous vehicles (AVs) to enhance traffic flow and efficiency may be balanced by the prospect of higher road usage as commuting becomes more convenient and attractive. Such intensification of traffic may result in congestion at intersections along primary thoroughfares, particularly during periods of peak traffic volume. The capacity of the infrastructure to accommodate the increased traffic volume, and thus maintain high efficiency, constitutes a significant challenge in these scenarios. Despite the inherent efficiency of AVs, congestion at critical points can still result from the sheer volume of vehicles, thereby underscoring the necessity for comprehensive urban planning and infrastructure development to accommodate the increased demand. Furthermore, the integration of AVs with public transport and the promotion of alternative commuting methods can assist in addressing these challenges and ensuring sustainable traffic management.

## 4. Conclusions

This study focuses on modeling urban traffic flow using micro-traffic simulation based on autonomous vehicles. It investigates the variations in urban traffic efficiency under different proportions of autonomous vehicles in the traffic flow. Previous research on traffic modeling using autonomous vehicles has mainly focused on modeling independent intersections or individual road segments. However, there is currently a lack of research on the impact of different proportions of autonomous vehicles on traffic efficiency within cities, and the conclusions drawn are relatively limited. Microsimulation can provide more comprehensive information and can guide the future configuration of autonomous vehicles in urban areas.

Based on the results of the impact of autonomous vehicles on urban traffic efficiency, this study explores the changes in commuting demand in small and medium-sized cities under the background of autonomous vehicles from the perspective of population and travel mode. However, due to constraints in research time, technology, and the number of articles, this article also has the following limitations:

This study also attempted to model the communication behavior of autonomous vehicles, specifically V2X. However, due to technological limitations, it was difficult to simulate the entire urban environment. Although the simulation included a large number of autonomous vehicles, they have not yet been implemented on a large scale in real-world roads, and people’s transportation preferences are unpredictable, leading to some deviations. The spatial distribution of urban traffic and employment mutually influence and interact with each other, creating a dynamic process. However, this study only predicts static time points. In future work, exploring patterns of changing traffic demand through a series of feedback loops can enable autonomous vehicles to better meet transportation needs. In addition, autonomous vehicles are not just vehicles with transportation capabilities but also crucial carriers of big data. They play a key role in achieving smart mobility, intelligent transportation, and the development of smart cities. Therefore, utilizing autonomous vehicles to meet China’s transportation needs is an important component of building smart cities in the future.

## Figures and Tables

**Table 1:** Parameters of the driver model used in SUMO simulations in this study.

Type	Mingap (m)	Accel (m/s^2^)	Decel (m/s^2^)	Emergency Decel (m/s^2^)	Sigma	Time Headway (s)
Unmanned vehicle	1.5	3	4	9	0	1
Human-driven vehicle	0.5	2.6	4.5	9	0.3	0.6

**Table 2:** Variables and evaluation indicators.

Type of Variable	Variables	Evaluation Indicators and Penetration Rate Values
Dependent variable	Urban transport efficiency	Average travel distance
		Average delay time
		Average waiting time
		Average travel time
		Average travel speed
Independent variables	Percentage of unmanned vehicles in the traffic flow	5–100%

**Table 3:** Road network modeling results.

Type	Travel Time (s)	Travel Distance (m)	Waiting Time (s)	Delay Time (s)	Travel Speed (m/s)
Manual driving vehicles	583.82	6543.22	101.49	193.31	11.21
Autonomous vehicle	526.81	6638.54	95.35	217.27	12.6
Average	544.65	6513.33	101.51	231.95	11.95

**Table 4:** Average queue length at Intersection 1.

Share of Autonomous Vehicle in Traffic Flow	North Side (m)			East Side (m)		
	Left	Straight	Straight	Left	Straight	Straight
90%	304.07	75.32	4.13	3.9	5.88	2.51
0%	791.4	218.69	19.89	6.54	10.69	3.15

**Table 5:** Average queue length at Intersection 2.

Share of Autonomous Vehicle in Traffic Flow	North Side (m)			East Side (m)	South Side (m)		
	Left	Straight	Straight	Left	Straight	Straight	Right
90%	105.86	19.05	9.97	6.34	49.64	37.06	34.71
0%	131.07	161.02	112.53	120.96	61.33	62.11	36.47

**Table 6:** Average queue length at Intersection 3.

Share of Autonomous Vehicle in Traffic Flow	North Side (m)		West Side (m)			South Side (m)		
	Straight	Straight	Straight	Left	Right	Left	Straight	Straight
90%	57.6	21.1	15.96	0	1.69	342.87	42.02	6.26
0%	15.96	43.92	50.85	0	3.79	280.1	36.07	7.01

**Table 7:** Average queue length for north-south direction.

Share of Autonomous Vehicle in Traffic Flow	North Side (m)			South Side (m)		
	Left	Straight	Straight	Left	Straight	Straight
90%	2.06	46.47	4.45	2.67	19.12	7.00
0%	2.76	175.77	118.86	2.11	33.71	4.88

**Table 8:** Average queue length for east-west direction.

Share of Autonomous Vehicle in Traffic Flow	East Side (m)			West Side (m)		
	Left	Straight	Straight	Left	Straight	Straight
90%	15.75	22.32	1.25	85.07	81.63	24.11
0%	54.77	36.55	1.90	124.89	122.18	25.87

## Data Availability

The historical data of passenger flow volume used in this paper is confidential data. Therefore, the data can only be provided after anonymization.
